# Psoriasiform Drug Eruption Associated with Sodium Valproate

**DOI:** 10.1155/2013/823469

**Published:** 2013-11-13

**Authors:** Gulen Gul Mert, Faruk Incecik, Suhan Gunasti, Ozlem Herguner, Sakir Altunbasak

**Affiliations:** ^1^Division of Pediatric Neurology, Department of Pediatrics, Cukurova University, 01180 Adana, Turkey; ^2^Department of Dermatology, Cukurova University, 01180 Adana, Turkey

## Abstract

As psoriasis is a common skin disorder, knowledge of the factors that may induce, trigger, or exacerbate the disease is of primary importance in clinical practice. Drug intake is a major concern in this respect, as new drugs are constantly being added to the list of factors that may influence the course of the disease. We report a patient with a psoriasiform drug eruption associated with the use of sodium valproate. Physicians should be aware of this type of reaction. Early detection of these cases has practical importance since the identification and elimination of the causative drug are essential for therapy success.

## 1. Introduction

Valproic acid (VPA), a branched chain aliphatic carboxylic acid, is a broad spectrum antiepileptic drug commonly used in childhood seizures, with good response rates and acceptable toxicity. It acts by multiple mechanisms like prolongation of sodium channel, attenuation of calcium-mediated “T” current, and augmentation of release of inhibitory transmitter GABA by inhibiting its degradation as well as probably increasing its synthesis. Oral absorption is good and it is excreted by urine [1]. Side effects include teratogenicity, weight gain, amenorrhea, alopecia, nausea, tremor, increased bone turnover, and rarely severe hepatotoxicity and pancreatitis [2]. About 10% of patients receiving antiepileptic drug therapy develop skin allergy. Among the antiepileptic drugs, valproic acid is relatively free from skin allergy [3]. We report a patient with a psoriasiform drug eruption associated with the use of sodium valproate.

## 2. Case

A 16-year-old boy who had epilepsy and mental retardation secondary to hypoxic ischemic encephalopathy was put on sodium valproate 500 mg twice a day for generalized tonic-clonic seizures. Approximately three months after starting sodium valproate, he presented with a psoriasiform eruption on his limbs and trunk. He had not taken any other medication, and he had no personal or family history of psoriasis. Routine laboratory investigations including complete blood cell count, liver and renal function tests, urinalysis were within normal limits.

**Figure 1:**
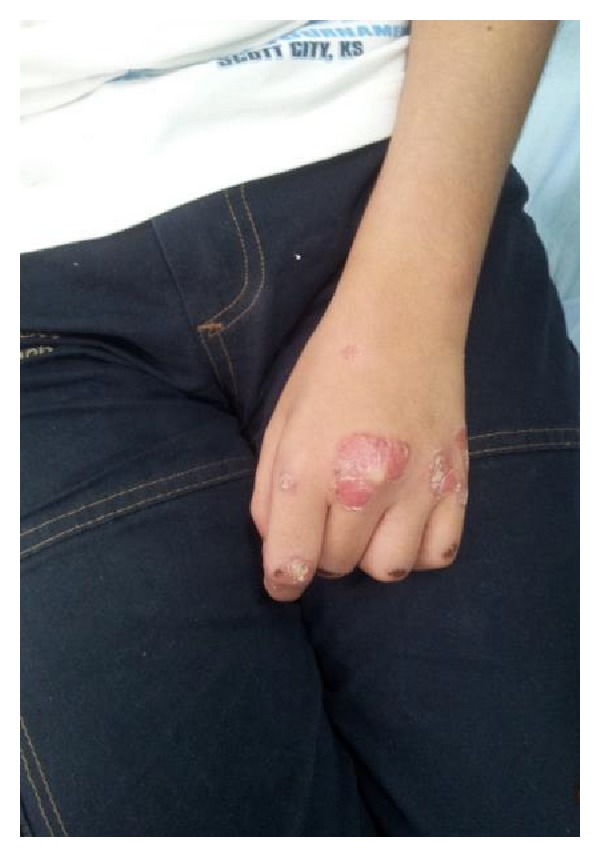
Psoriasiform lesions.

**Figure 2:**
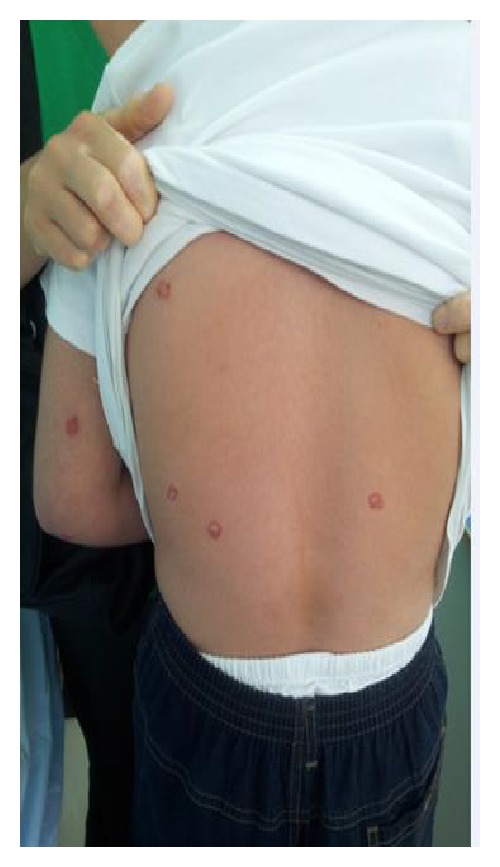
Psoriasiform lesions.

**Figure 3:**
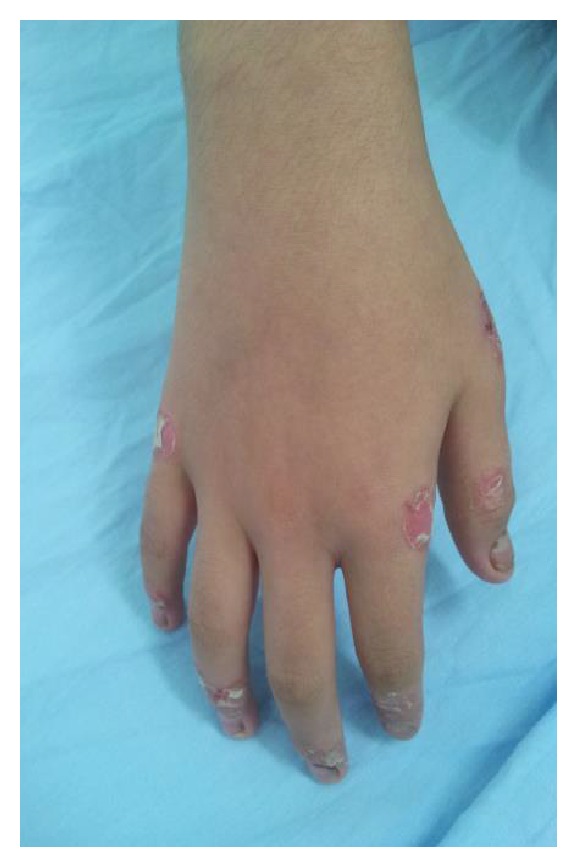
Psoriasiform lesions.

On dermatologic examination he had erythematous white scaly, sharply bordered plaques and papules on back of his hands, upper limbs, and posterior trunk. The morphology of the lesions was suggestive of psoriasiform eruption (Figures 1, 2, and 3). 

**Figure 4:**
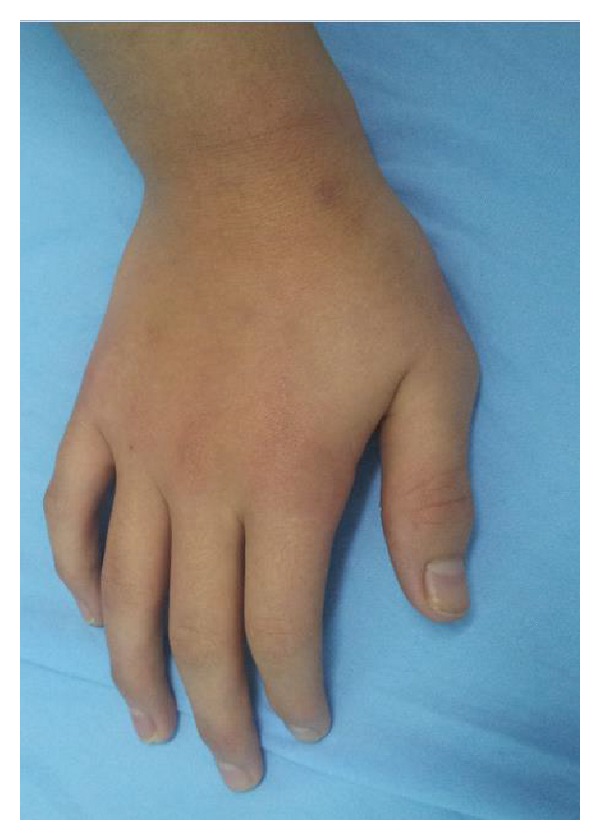
Recovery after drug cessation.

**Figure 5:**
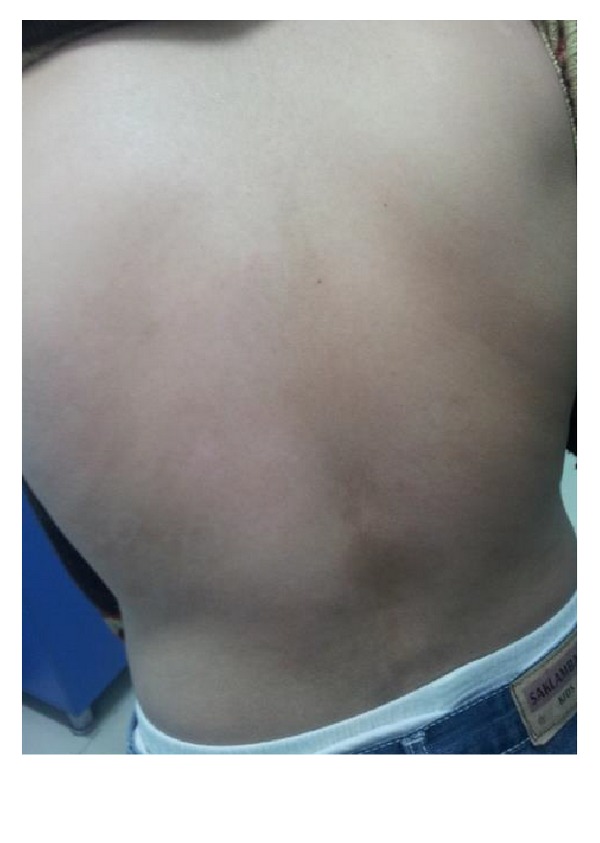
Recovery after drug cessation.

After discontinuation of sodium valproate, the skin lesions disappeared within 4 weeks without any oral or topical treatment. No relapse of the cutaneous eruption was observed during one year following cessation of sodium valproate (Figures 4 and 5).

Development of psoriasiform eruption with the initiation of valproate and subsequent remission of the lesions with discontinuation of the drug and subsequent course clearly suggests a casual relation between sodium valproate and skin lesions. 

## 3. Discussion

Cutaneous drug eruptions associated with antiepileptic drugs (AEDs) can range from maculopapular eruption to severe Stevens-Johnson syndrome or toxic epidermal necrolysis. The aromatic drugs phenytoin, carbamazepine, oxcarbazepine, phenobarbital, primidone, zonisamide, and lamotrigine are the most common offenders. In contrast, the second generation AEDs like valproate, topiramate, gabapentin, tiagabine, and levetiracetam are rarely associated with rash. Cutaneous eruptions were reported with valproate compounds [4]. Review of the literature showed only three cases of psoriasiform eruption with valproate [5, 6].

The pathogenesis of drug-related psoriasiform eruptions or exacerbation of preexisting psoriasis remains unclear. Delayed hypersensitivity, impaired lymphocyte transformation, and decreases in epidermal cyclic adenosine monophosphate (cAMP) have been proposed as causes for these unusual drug reactions [7]. Rapid and complete clearing of the lesions after cessation of the drug and an absence of relapse after clearing allowed us to conclude that valproate was the cause of the psoriasiform eruption.

Psoriasis is a common skin disorder with unknown etiology which may be induced or triggered by medications, infections, and stress. Some of the most common medications known to trigger or worsen psoriasis include lithium, gold salts, beta blockers, and antimalarials. Many other drugs have also been suspected to trigger psoriasis. There are two variants of drug provoked psoriasis: drug induced psoriasis in which discontinuation of the drug will stop further progression of the disease and drug triggered psoriasis in which the disease progresses even after discontinuation of the drug [7, 8]. In our patient, the disease had completely resolved after discontinuation of the drug.

The number of prescribed medications per patient per year is increasing, and since many medications may cause psoriasiform eruptions, it is important for physicians to recognize this condition.
